# An automatic method for myocardial T2* curve fitting in thalassemia patients with severe iron overload

**DOI:** 10.1186/1532-429X-15-S1-E31

**Published:** 2013-01-30

**Authors:** Antonella Meloni, Vincenzo Positano, Petra Keilberg, Maria Filomena Santarelli, Luigi Landini, Giuseppina Secchi, Carmelo Fidone, Maria Antonietta Romeo, Letizia Gulino, Massimo Lombardi, Alessia Pepe

**Affiliations:** 1 CMR Unit, Fondazione G.Monasterio CNR-Regione Toscana and Institute of Clinical Physiology, Pisa, Italy; 2 Servizio trasfusionale, Azienda USL n° 1, Sassari, Italy; 3 U.O.S. di Microcitemia, Az. Osp. Civile, Ragusa, Italy; 4 Dipartimento Pediatria, Azienda Policlinico, Catania, Italy

## Background

Myocardial iron overload assessment by multislice multiecho T2* technique is used in the clinical management of thalassemia major (TM) patients. Signal decay curves are extracted from the 16 left ventricular (LV) segments and the fitting of these curves to a mono-exponential model provides the corresponding T2* values. In patients with severe cardiac iron overload, where signal will decay quickly becoming comparable to image noise, manual truncation of signal decay curves excluding later echo times (TEs) is adopted. In this study an automatic truncation method avoiding the variability associated with the manual selection of the truncation point is introduced and validated.

## Methods

Twenty patients (13 males, age 33±7 years) enrolled in the MIOT Network and diagnosed for severe iron overload (T2*<10 ms) were considered. Using a previously validated software the segmental T2* values were evaluated by the standard methodology (i.e. manual truncation).

Images were independently analysed by the developed automated approach. The percentage fitting error (e) was computed as the root mean square error (MRSE) between the signal decay curve and the mono-exponential model normalized to the mean value of the signal. If e was > 5%, the algorithm cut-off the last TE and performed again the fitting. The procedure was iterated until the error become <5% or the number of TEs become equal to three. To assess the inter-operator variability, the dataset was processed by a second operator.

## Results

**Figure 1:**
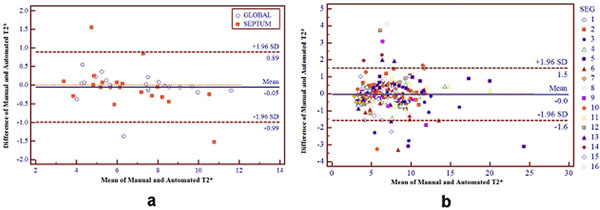


The Coefficient of Variability (CoV) for inter-observer variability was 6.82±4.01%. The CoV between automated and manual analysis was 6.15±3.92 %, not significantly different from inter-observer variability (P=0.332). No significant difference was detected between mid-septum and global T2* values evaluated with manual and automated procedure (P=0.26 and P=0.91, respectively). The mean fitting error was not significantly different in manual and automated analysis (4.10±2.11 vs. 4.52±2.12, P=0.53). In segmental analysis, no significant differences were found between manual and automatic procedure (P>0.01 for all segments). Figure 1 shows the Bland-Altmann plots for global heart and mid-ventricular septum T2* measurements (a) and for segmental T2* values (b).

## Conclusions

Truncation of signal decay curve needed to compensate for low signal in later echoes in patients with severe iron overload can be effectively automatized avoiding operator induced variability.

## Funding

The MIOT project receives "no-profit support" from industrial sponsorships (Chiesi and Apotex). This study was also supported by: "Ministero della Salute, fondi ex art. 12 D.Lgs. 502/92 e s.m.i., ricerca sanitaria finalizzata anno 2006" e "Fondazione L. Giambrone".

